# Supplementary material for: Cross-national analyses require additional controls to account for the non-independence of nations
Source: Nat Commun. 2023 Sep 18;14:5776. doi: 10.1038/s41467-023-41486-1 (PMC10507061; doi:10.1038/s41467-023-41486-1)
Supplement: Supplementary file 1 — Supplementary Information [file 41467_2023_41486_MOESM1_ESM.pdf]

Supplementary Information

Cross-national analyses require additional controls to account for the non-independence of nations

Scott Claessens[1], Thanos Kyritsis[1], & Quentin D. Atkinson[1,2]

[1] School of Psychology, University of Auckland, New Zealand

[2] School of Anthropology and Museum Ethnography, University of Oxford, United Kingdom

Supplementary Methods

Bayesian models for reanalysis. We provide model formulae for our reanalyses of cross-national correlations, for a general bivariate case with standardised outcome Y and predictor X variables. In the naive regression model without controls for non-independence:

$$
\begin{aligned}
Y_i &\sim \text{Normal}(\mu_i, \sigma) \\
\mu_i &= \alpha + \beta X_i \\
\alpha &\sim \text{Normal}(0, 0.4) \\
\beta &\sim \text{Normal}(0, 0.4) \\
\sigma &\sim \text{Exponential}(5)
\end{aligned}
\tag{S1}
$$

The priors in this model were arrived at by prior predictive checks, with wider priors making predictions beyond the scale of standardised outcome variables and narrower priors being too informative.

To control for spatial non-independence, we add a Gaussian process to this model and feed it a scaled geographic distance matrix D based on Euclidean distances between latitude and longitude coordinates. This distance matrix is computed internally by the *brms* R package[1]. The Gaussian process uses an exponentiated quadratic covariance kernel, the only covariance kernel currently supported by *brms*. The model formula is:

$$
\begin{aligned}
Y_i &\sim \text{Normal}(\mu_i, \sigma) \\
\mu_i &= \alpha + \kappa_{\text{NATION}[i]} + \beta X_i \\
\begin{pmatrix} \kappa_1 \\ \kappa_2 \\ \dots \\ \kappa_n \end{pmatrix} &\sim \text{MVNormal}\left(\begin{pmatrix} 0 \\ 0 \\ \dots \\ 0 \end{pmatrix}, \mathbf{K} \right) \\
\mathbf{K}_{ij} &= sdgp^2 \text{exp}\Big(- D_{ij}^2 / (2 lscale^2) \Big) \\
\alpha &\sim \text{Normal}(0, 0.4) \\
\beta &\sim \text{Normal}(0, 0.4) \\
\sigma &\sim \text{Exponential}(5) \\
sdgp &\sim \text{Exponential}(5) \\
lscale &\sim \text{InverseGamma}(?, ?)
\end{aligned}
\tag{S2}
$$

where n is the number of nations, and D_{ij}^2 reflects the squared Euclidean distances between latitude and longitude coordinates for the i-th and j-th nations. Notice that the inverse gamma prior on *lscale* is left undetermined. This is because the *brms* package intelligently tunes the prior for this parameter based on the covariates of the Gaussian process (see https://betanalpha.github.io/assets/case_studies/gp_part3/part3.html).

To control for cultural phylogenetic non-independence, we manually specify the covariance structure for nation random intercepts using a pre-computed linguistic proximity matrix L (see previous section). The covariance between two nations is assumed to be linearly proportional to the linguistic proximity between those nations. This assumption is justified if we assume that cultural traits evolve neutrally via Brownian motion along a language phylogeny. Although this is a conservative assumption, we follow this approach here rather

than another Gaussian Process for several reasons: (1) to avoid model non-convergence when later including a spatial Gaussian Process in the same model, (2) to work around the lack of a coordinate system for linguistic distances, as opposed to latitude and longitude values for geographic distance, and (3) to showcase different ways that researchers can allow nations to covary to control for non-independence.

The non-centered parameterisation of this model is:

$$
\begin{aligned}
Y_i &\sim \text{Normal}(\mu_i, \sigma) \\
\mu_i &= \alpha + z_{\text{NATION}[i]}\sigma_\alpha L + \beta X_i \\
\alpha &\sim \text{Normal}(0, 0.4) \\
\beta &\sim \text{Normal}(0, 0.4) \\
z_j &\sim \text{Normal}(0, 1) \\
\sigma_\alpha &\sim \text{Exponential}(5) \\
\sigma &\sim \text{Exponential}(5)
\end{aligned}
\tag{S3}
$$

Finally, we can control for spatial and cultural phylogenetic non-independence simultaneously by including both a Gaussian process over latitude and longitude coordinates *and* nation random intercepts that covary according to linguistic proximity. The resulting model is as follows:

$$
\begin{aligned}
Y_i &\sim \text{Normal}(\mu_i, \sigma) \\
\mu_i &= \alpha + \kappa_{\text{NATION}[i]} + z_{\text{NATION}[i]}\sigma_\alpha L + \beta X_i \\
\begin{pmatrix} \kappa_1 \\ \kappa_2 \\ \dots \\ \kappa_n \end{pmatrix} &\sim \text{MVNormal}\left(\begin{pmatrix} 0 \\ 0 \\ \dots \\ 0 \end{pmatrix}, \mathbf{K} \right) \\
\mathbf{K}_{ij} &= sdgp^2 \exp\Big(- D_{ij}^2/(2lscale^2) \Big) \\
\alpha &\sim \text{Normal}(0, 0.4) \\
\beta &\sim \text{Normal}(0, 0.4) \\
z_j &\sim \text{Normal}(0, 1) \\
\sigma_\alpha &\sim \text{Exponential}(5) \\
\sigma &\sim \text{Exponential}(5) \\
sdgp &\sim \text{Exponential}(5) \\
lscale &\sim \text{InverseGamma}(?, ?)
\end{aligned}
\tag{S4}
$$

Supplementary Figures

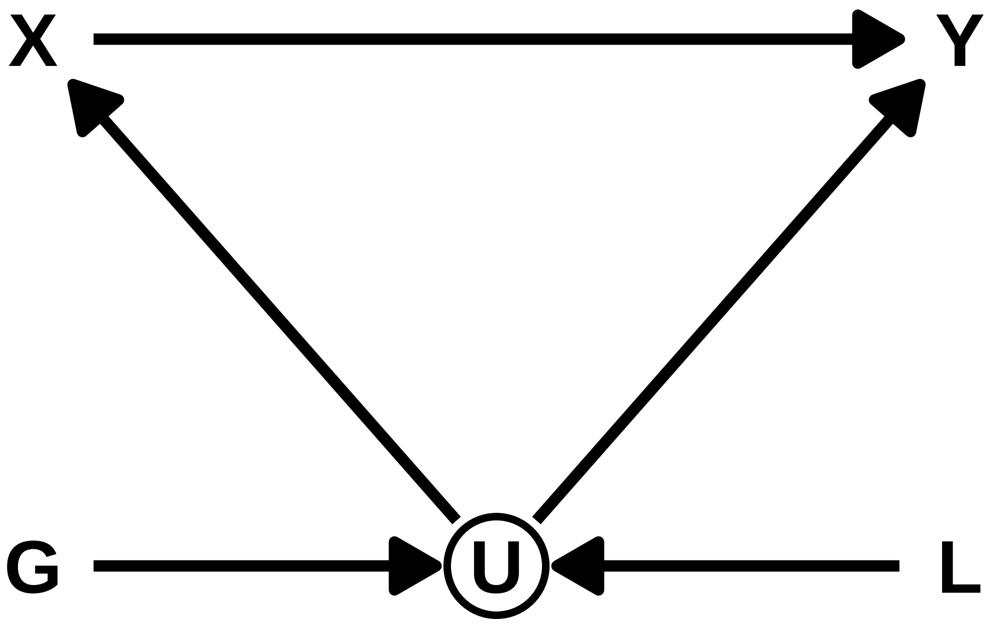

Supplementary Figure 1. A causal directed acyclic graph of spatial and cultural phylogenetic non-independence in cross-national studies. We are interested in estimating the direct effect of national-level exposure X on national-level outcome Y. But these variables are confounded by their common unobserved cause U. U is a stand-in for shared environmental, ecological, and geographic causes (e.g. climate, biodiversity, physical topography) and cultural and institutional causes (e.g. cultural norms, technologies, and institutions). In this causal model, we need to condition on U to estimate the direct path from X to Y, but we cannot since it is unobserved. However, geographic G and linguistic L relationships between societies influence U, since changing a nation's spatial distance to or shared cultural ancestry with other nations will change its environmental and cultural traits. We can thus use G and L to model the covariation between X and Y induced by U. Failing to do this and simply estimating the bivariate correlation between X and Y will produce spurious relationships and residuals that are spatially and culturally non-independent around the world.

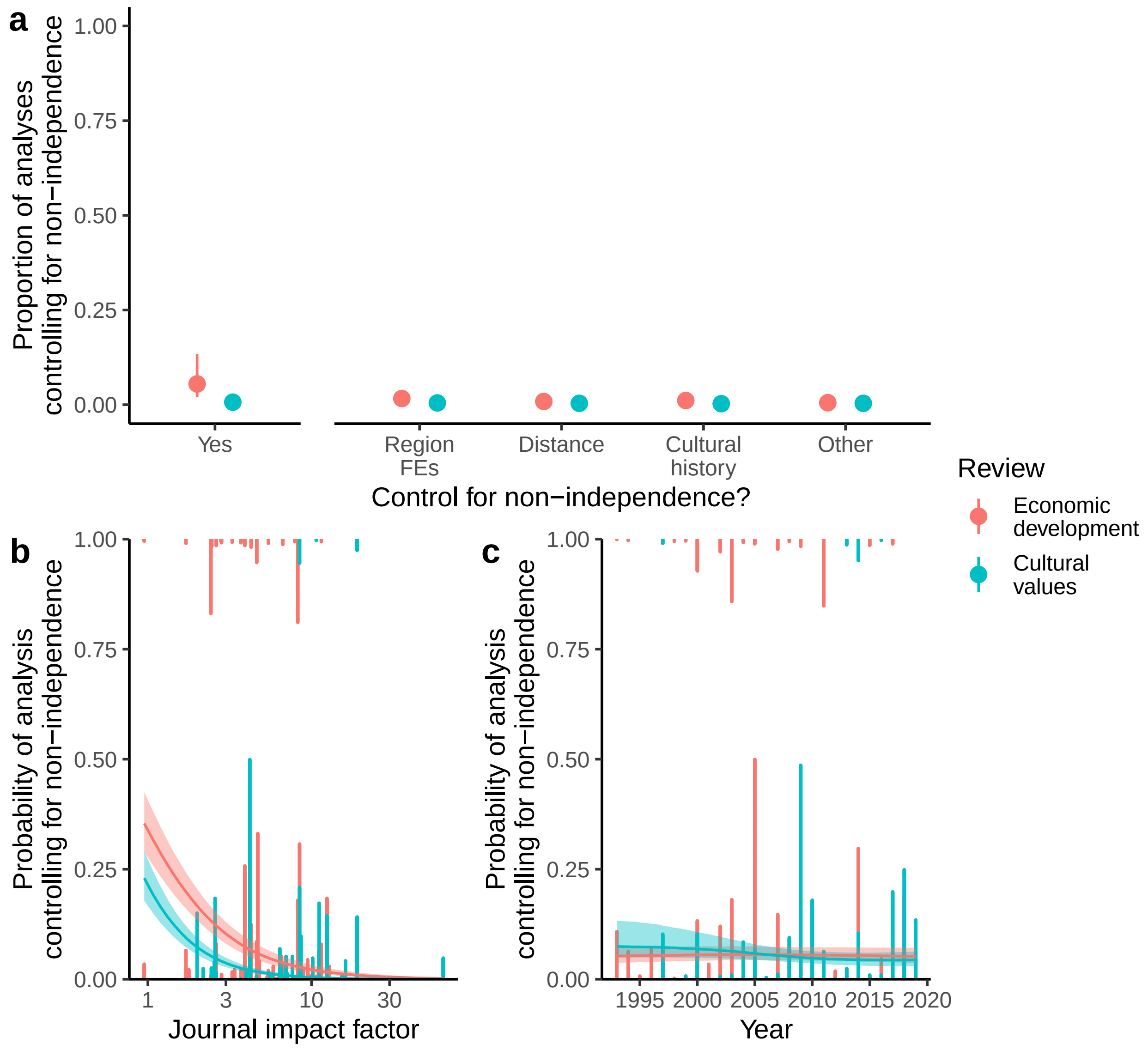

Supplementary Figure 2. *Analysis-level results from literature review of 100 highly-cited cross-national studies of economic development (red) and cultural values (blue).* (a) Proportion of analyses accounting for non-independence, overall and split by common methods of controlling for non-independence. Points represent posterior median proportions and ranges represent equal-tailed 95% credible intervals (n = 4308 observations). (b) The association between journal impact factor and the probability that an analysis accounts for non-independence. (c) Estimated trend over time for the probability that an analysis accounts for non-independence. Lines and shaded areas are posterior median regression lines and equal-tailed 50% credible intervals from Bayesian multilevel models (n = 4308 observations). Histograms represent relative counts for individual analyses that did (top) or did not (bottom) account for non-independence. Region FEs = region fixed effects.

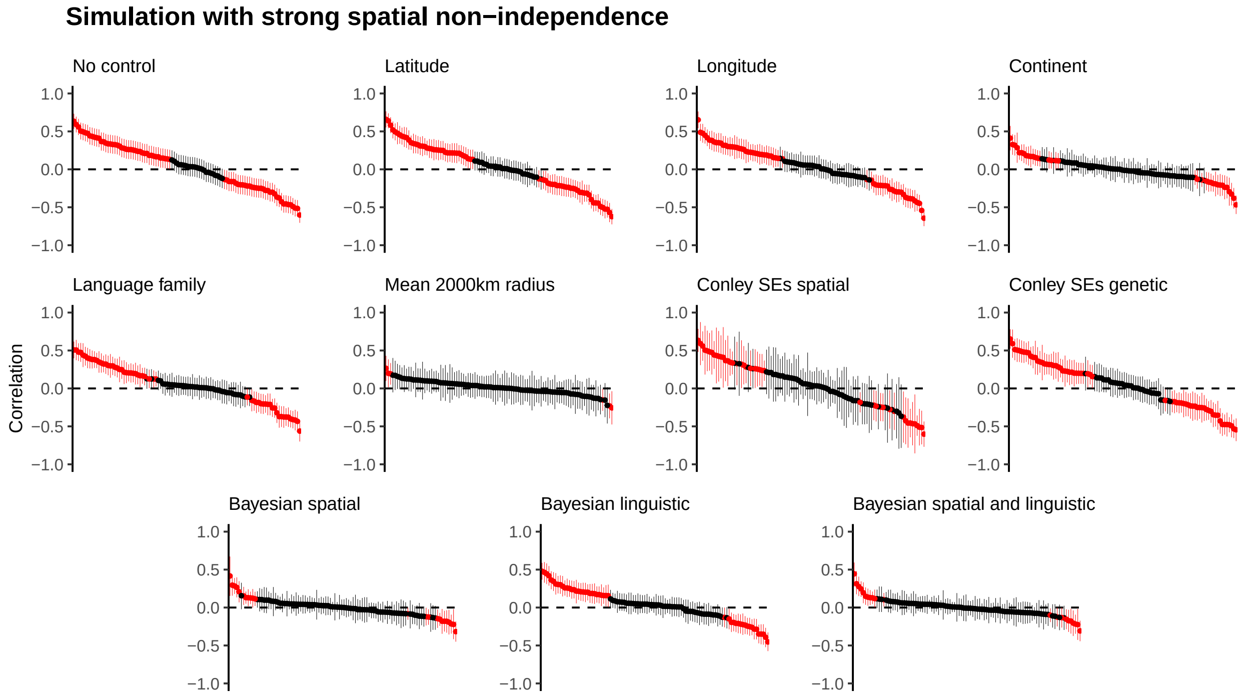

Supplementary Figure 3. Distribution of cross-national correlations from simulation study under strong spatial autocorrelation. In these simulations, the strength of spatial autocorrelation is set to 0.8 for both outcome and predictor variables, and the true correlation is set to 0. For frequentist regression models, points represent correlation estimates and ranges represent two-tailed 95% confidence intervals (n = 236 observations). For Bayesian regression models, points represent posterior means and ranges represent equal-tailed 95% credible intervals (n = 236 observations). Correlations are ordered by effect size independently in each panel. Red point ranges indicate that the 95% confidence / credible interval excludes zero. Black point ranges indicate that the 95% confidence / credible interval includes zero. SEs = standard errors.

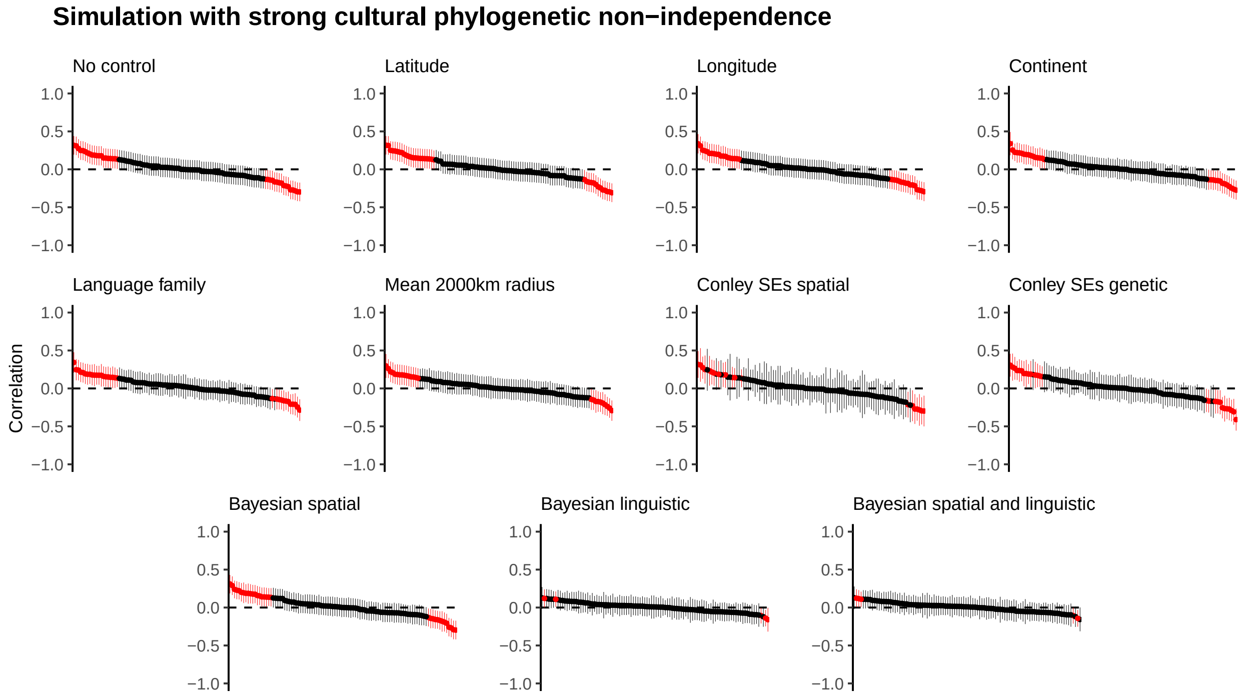

Supplementary Figure 4. Distribution of cross-national correlations from simulation study under strong cultural phylogenetic autocorrelation. In these simulations, the strength of cultural phylogenetic autocorrelation is set to 0.8 for both outcome and predictor variables, and the true correlation is set to 0. For frequentist regression models, points represent correlation estimates and ranges represent two-tailed 95% confidence intervals (n = 236 observations). For Bayesian regression models, points represent posterior means and ranges represent equal-tailed 95% credible intervals (n = 236 observations). Correlations are ordered by effect size independently in each panel. Red point ranges indicate that the 95% confidence / credible interval excludes zero. Black point ranges indicate that the 95% confidence / credible interval includes zero. SEs = standard errors.

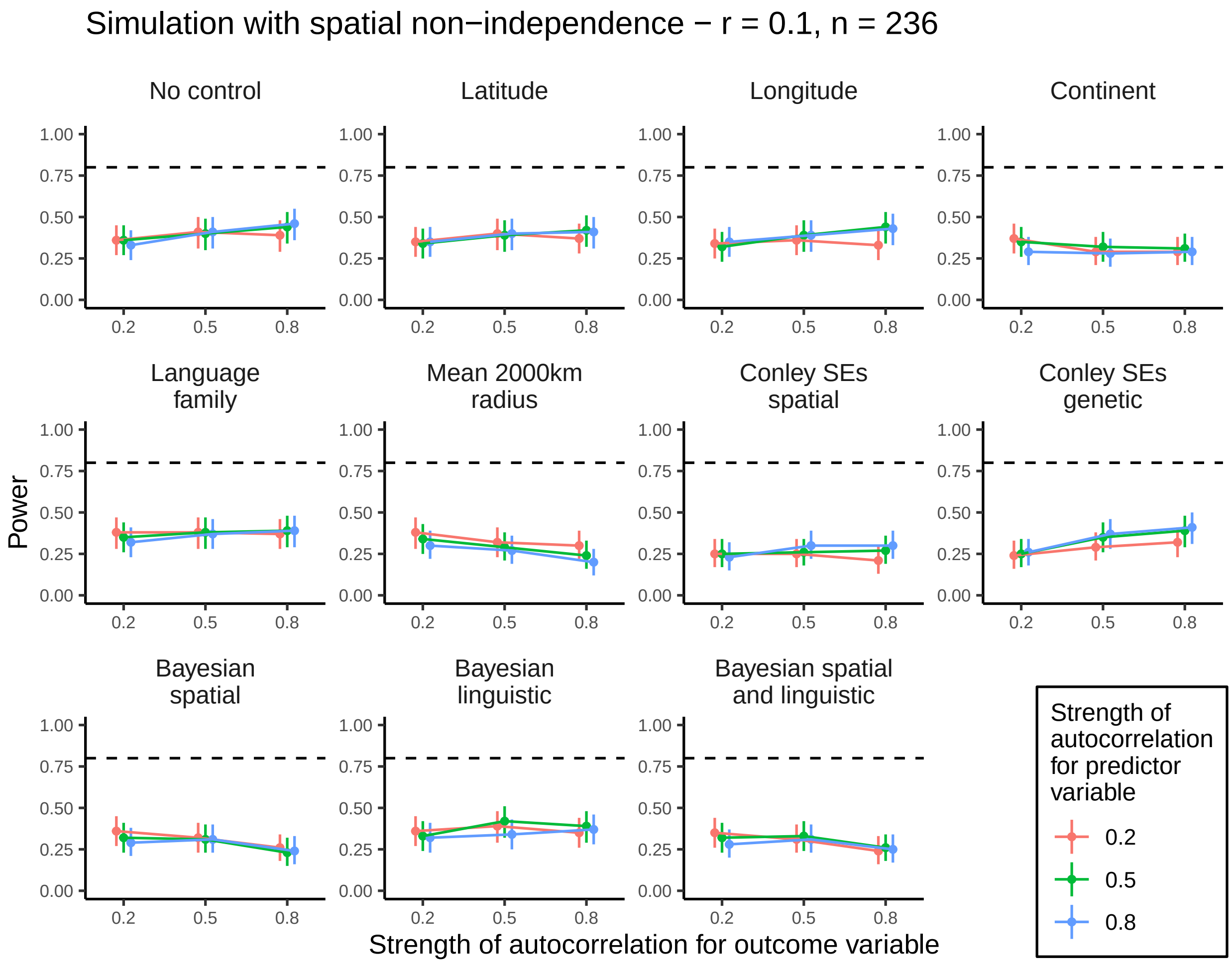

Supplementary Figure 5. *Statistical power estimates for different methods of controlling for spatial non-independence in our simulation study, assuming a small true effect size (r = 0.1).* For simulated outcome and predictor variables, we systematically varied the strength of spatial autocorrelation, from weak (0.2) to moderate (0.5) to strong (0.8). We simulated 100 datasets per parameter combination assuming a small true correlation between variables ($r = 0.1$) and fitted different models to each dataset. Statistical power was operationalised as the proportion of models that estimated a slope with a two-tailed 95% confidence / credible interval excluding zero. Points represent raw proportions of models with slope 95% confidence / credible intervals excluding zero, ranges represent two-tailed 95% bootstrap confidence intervals (n = 1000 bootstrap samples), and dashed lines indicate 80% power. Colours indicate whether the strength of autocorrelation for the predictor variable is 0.2 (red), 0.5, (green) or 0.8 (blue). SEs = standard errors.

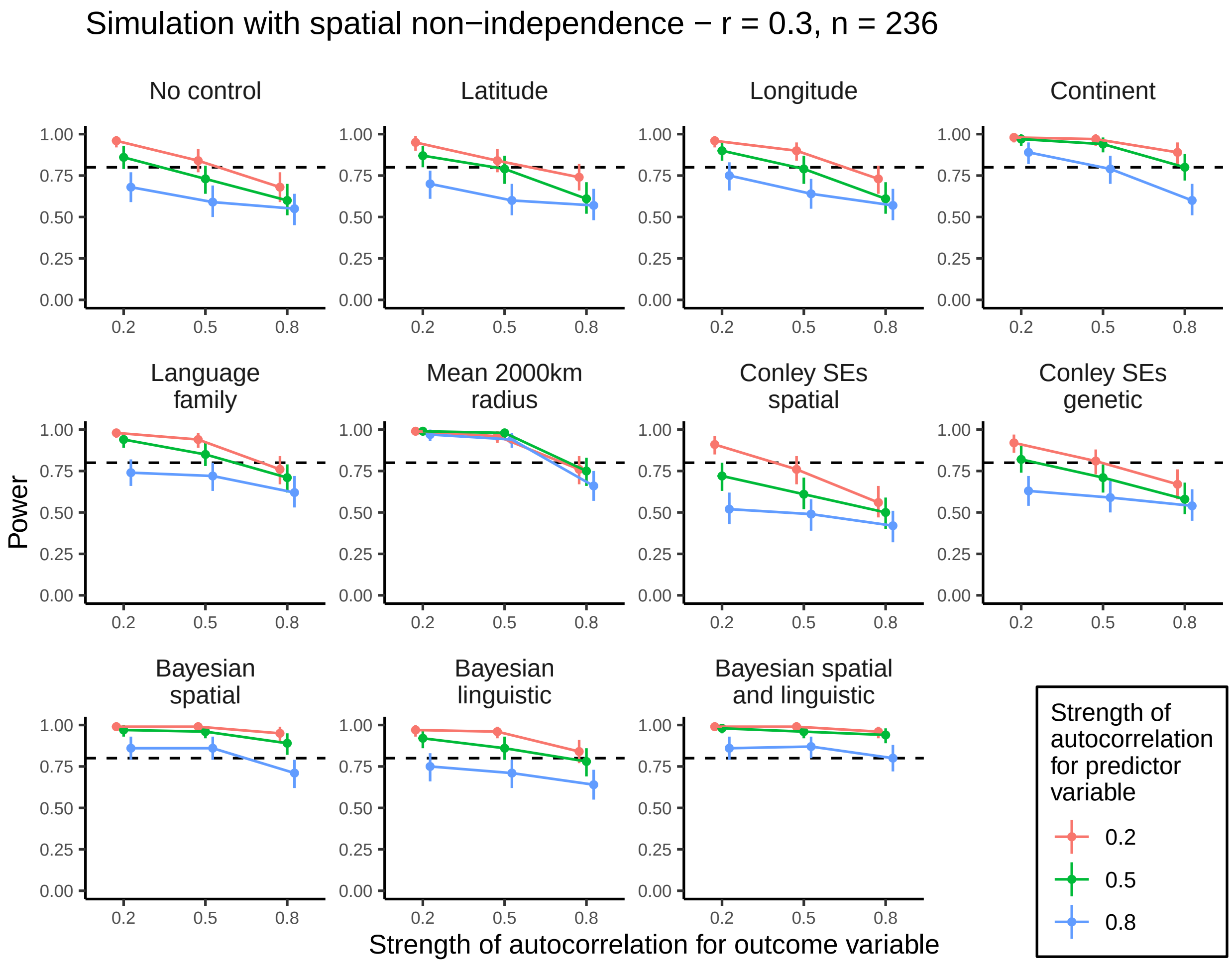

Supplementary Figure 6. *Statistical power estimates for different methods of controlling for spatial non-independence in our simulation study, assuming a medium true effect size (r = 0.3).* For simulated outcome and predictor variables, we systematically varied the strength of spatial autocorrelation, from weak (0.2) to moderate (0.5) to strong (0.8). We simulated 100 datasets per parameter combination assuming a medium true correlation between variables ($r = 0.3$) and fitted different models to each dataset. Statistical power was operationalised as the proportion of models that estimated a slope with a two-tailed 95% confidence / credible interval excluding zero. Points represent raw proportions of models with slope 95% confidence / credible intervals excluding zero, ranges represent two-tailed 95% bootstrap confidence intervals (n = 1000 bootstrap samples), and dashed lines indicate 80% power. Colours indicate whether the strength of autocorrelation for the predictor variable is 0.2 (red), 0.5, (green) or 0.8 (blue). SEs = standard errors.

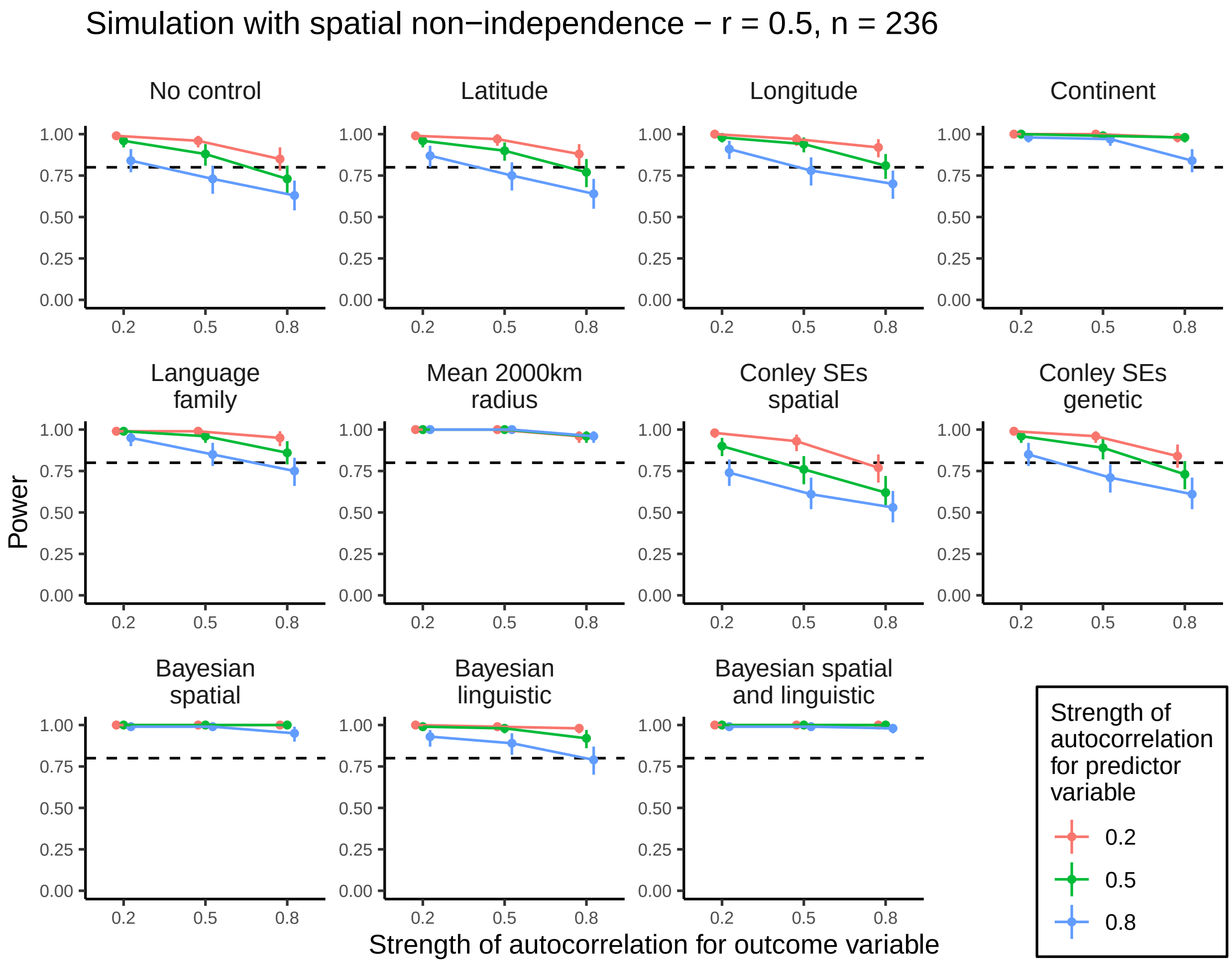

Supplementary Figure 7. Statistical power estimates for different methods of controlling for spatial non-independence in our simulation study, assuming a large true effect size (r = 0.5). For simulated outcome and predictor variables, we systematically varied the strength of spatial autocorrelation, from weak (0.2) to moderate (0.5) to strong (0.8). We simulated 100 datasets per parameter combination assuming a large true correlation between variables ($r = 0.5$) and fitted different models to each dataset. Statistical power was operationalised as the proportion of models that estimated a slope with a two-tailed 95% confidence / credible interval excluding zero. Points represent raw proportions of models with slope 95% confidence / credible intervals excluding zero, ranges represent two-tailed 95% bootstrap confidence intervals (n = 1000 bootstrap samples), and dashed lines indicate 80% power. Colours indicate whether the strength of autocorrelation for the predictor variable is 0.2 (red), 0.5, (green) or 0.8 (blue). SEs = standard errors.

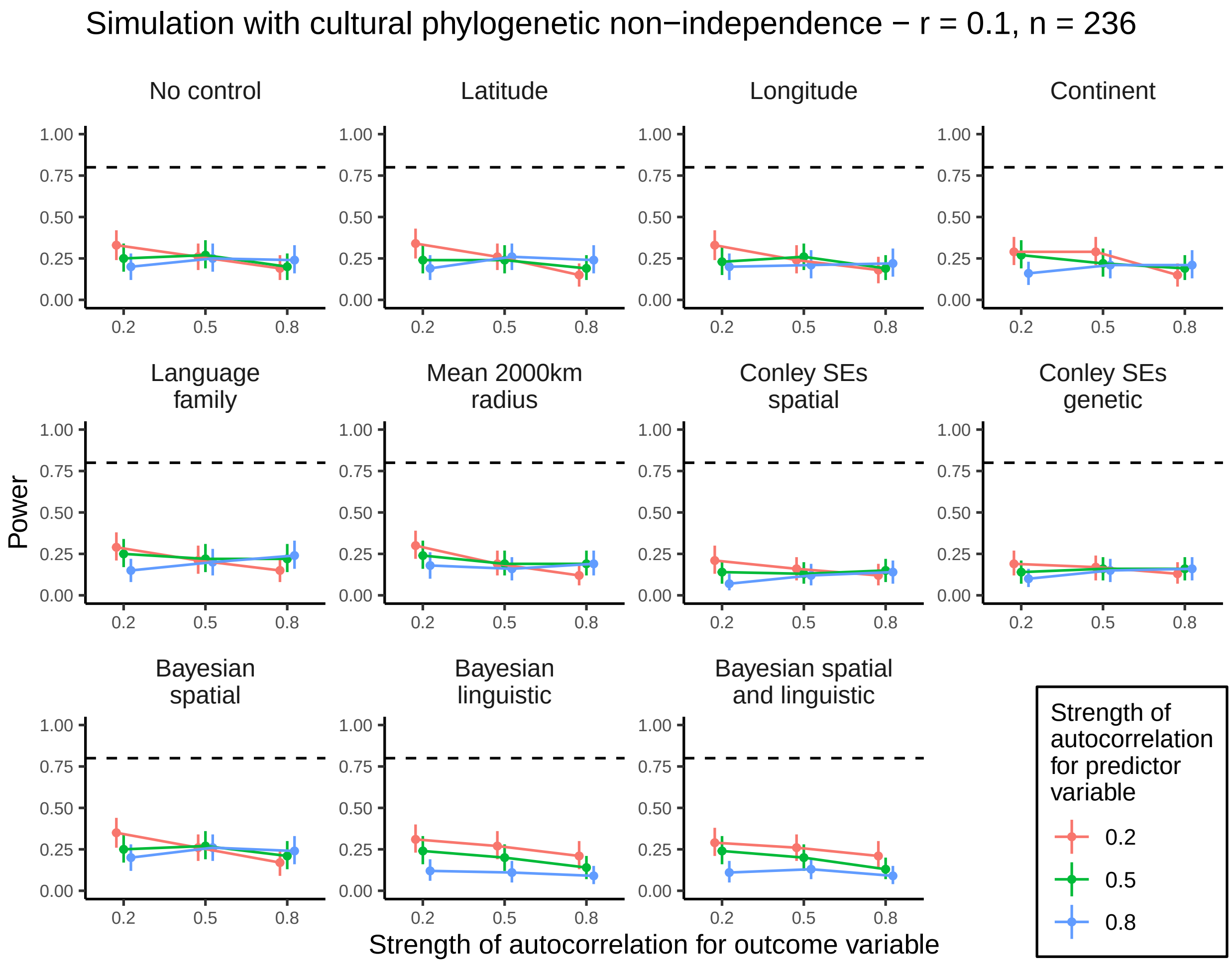

Supplementary Figure 8. *Statistical power estimates for different methods of controlling for cultural phylogenetic non-independence in our simulation study, assuming a small true effect size (r = 0.1).* For simulated outcome and predictor variables, we systematically varied the strength of cultural phylogenetic autocorrelation, from weak (0.2) to moderate (0.5) to strong (0.8). We simulated 100 datasets per parameter combination assuming a small true correlation between variables ($r = 0.1$) and fitted different models to each dataset. Statistical power was operationalised as the proportion of models that estimated a slope with a two-tailed 95% confidence / credible interval excluding zero. Points represent raw proportions of models with slope 95% confidence / credible intervals excluding zero, ranges represent two-tailed 95% bootstrap confidence intervals (n = 1000 bootstrap samples), and dashed lines indicate 80% power. Colours indicate whether the strength of autocorrelation for the predictor variable is 0.2 (red), 0.5, (green) or 0.8 (blue). SEs = standard errors.

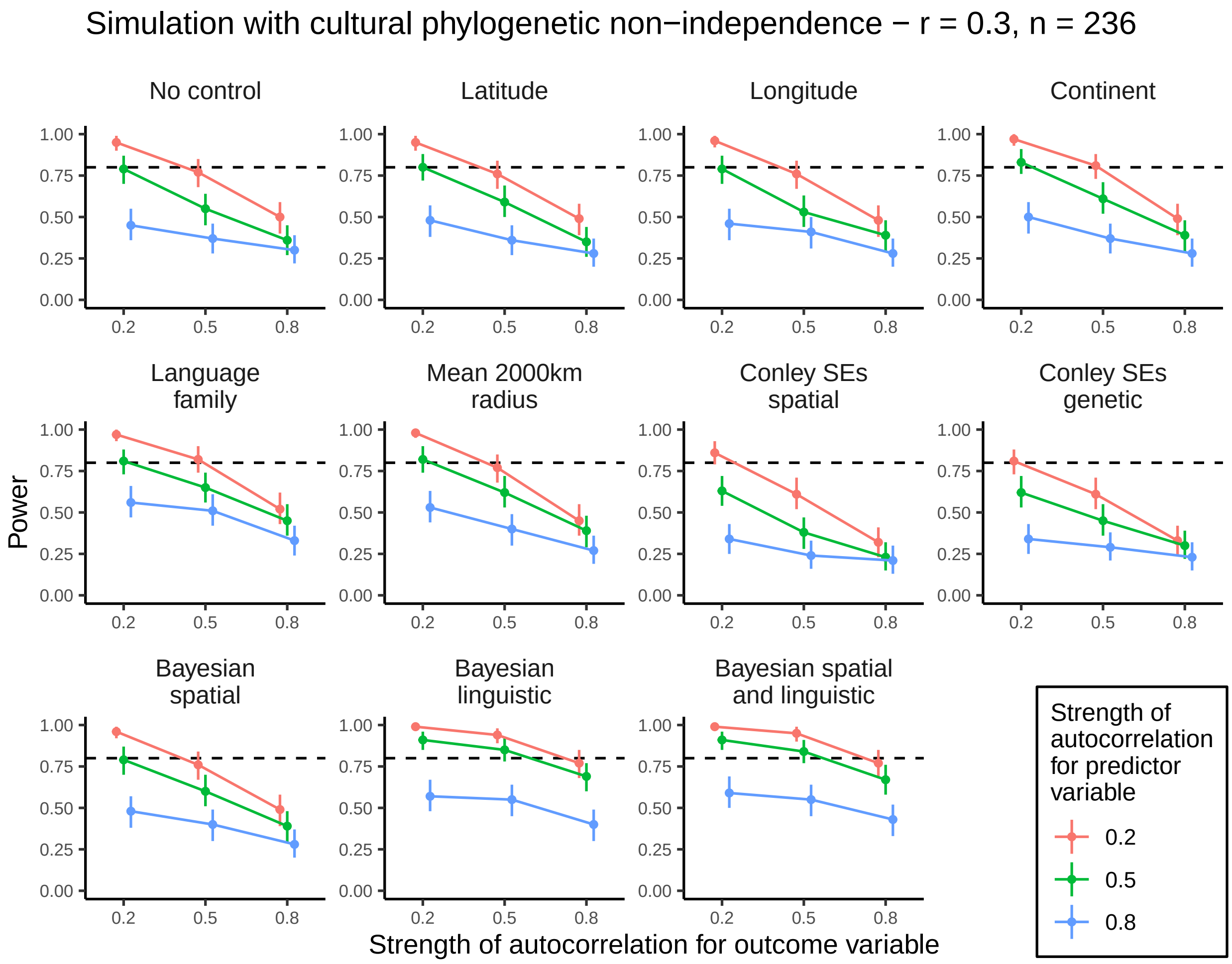

Supplementary Figure 9. Statistical power estimates for different methods of controlling for cultural phylogenetic non-independence in our simulation study, assuming a medium true effect size (r = 0.3). For simulated outcome and predictor variables, we systematically varied the strength of cultural phylogenetic autocorrelation, from weak (0.2) to moderate (0.5) to strong (0.8). We simulated 100 datasets per parameter combination assuming a medium true correlation between variables ($r = 0.3$) and fitted different models to each dataset. Statistical power was operationalised as the proportion of models that estimated a slope with a two-tailed 95% confidence / credible interval excluding zero. Points represent raw proportions of models with slope 95% confidence / credible intervals excluding zero, ranges represent two-tailed 95% bootstrap confidence intervals (n = 1000 bootstrap samples), and dashed lines indicate 80% power. Colours indicate whether the strength of autocorrelation for the predictor variable is 0.2 (red), 0.5, (green) or 0.8 (blue). SEs = standard errors.

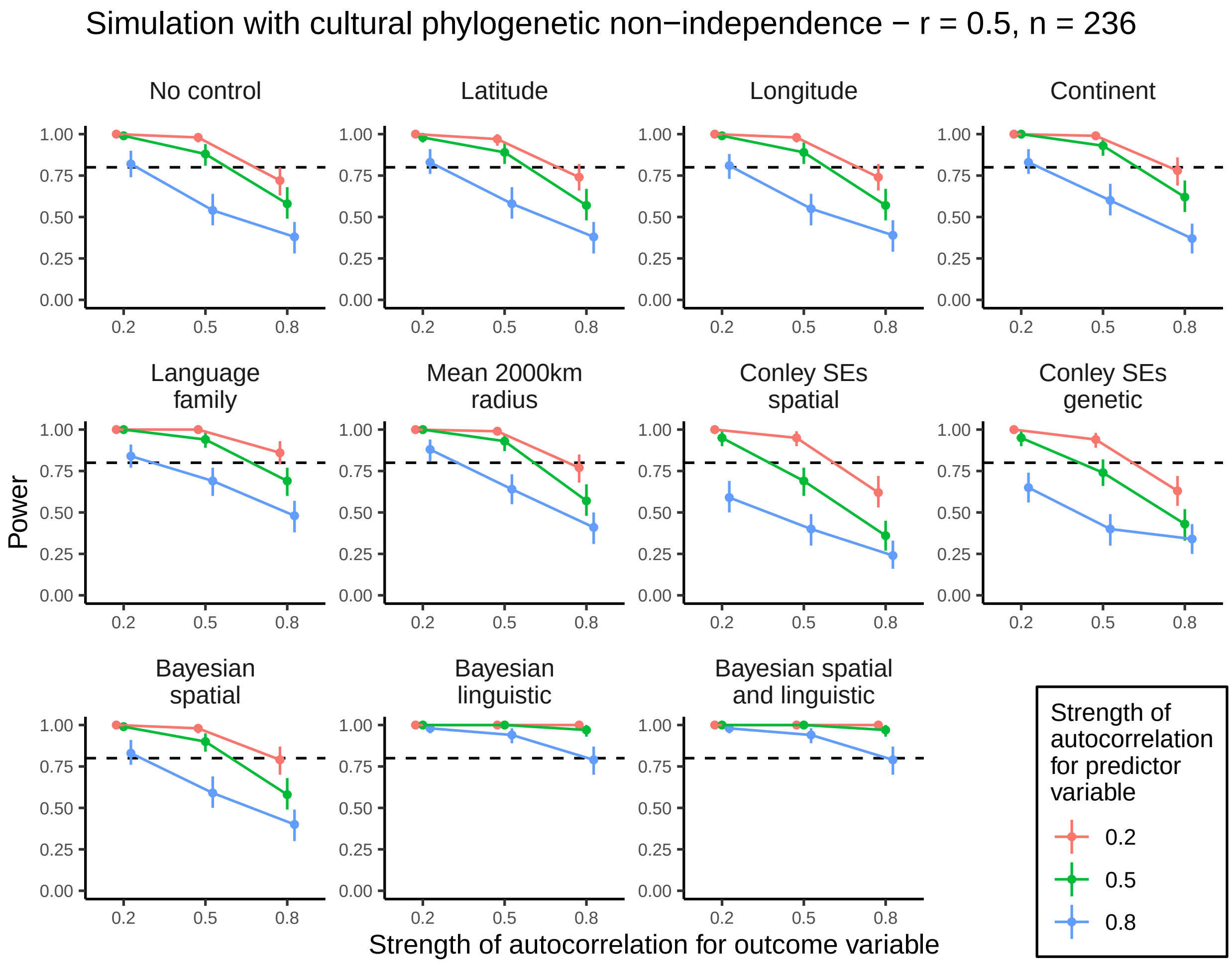

Supplementary Figure 10. Statistical power estimates for different methods of controlling for cultural phylogenetic non-independence in our simulation study, assuming a large true effect size (r = 0.5). For simulated outcome and predictor variables, we systematically varied the strength of cultural phylogenetic autocorrelation, from weak (0.2) to moderate (0.5) to strong (0.8). We simulated 100 datasets per parameter combination assuming a large true correlation between variables ($r = 0.5$) and fitted different models to each dataset. Statistical power was operationalised as the proportion of models that estimated a slope with a two-tailed 95% confidence / credible interval excluding zero. Points represent raw proportions of models with slope 95% confidence / credible intervals excluding zero, ranges represent two-tailed 95% bootstrap confidence intervals (n = 1000 bootstrap samples), and dashed lines indicate 80% power. Colours indicate whether the strength of autocorrelation for the predictor variable is 0.2 (red), 0.5, (green) or 0.8 (blue). SEs = standard errors.

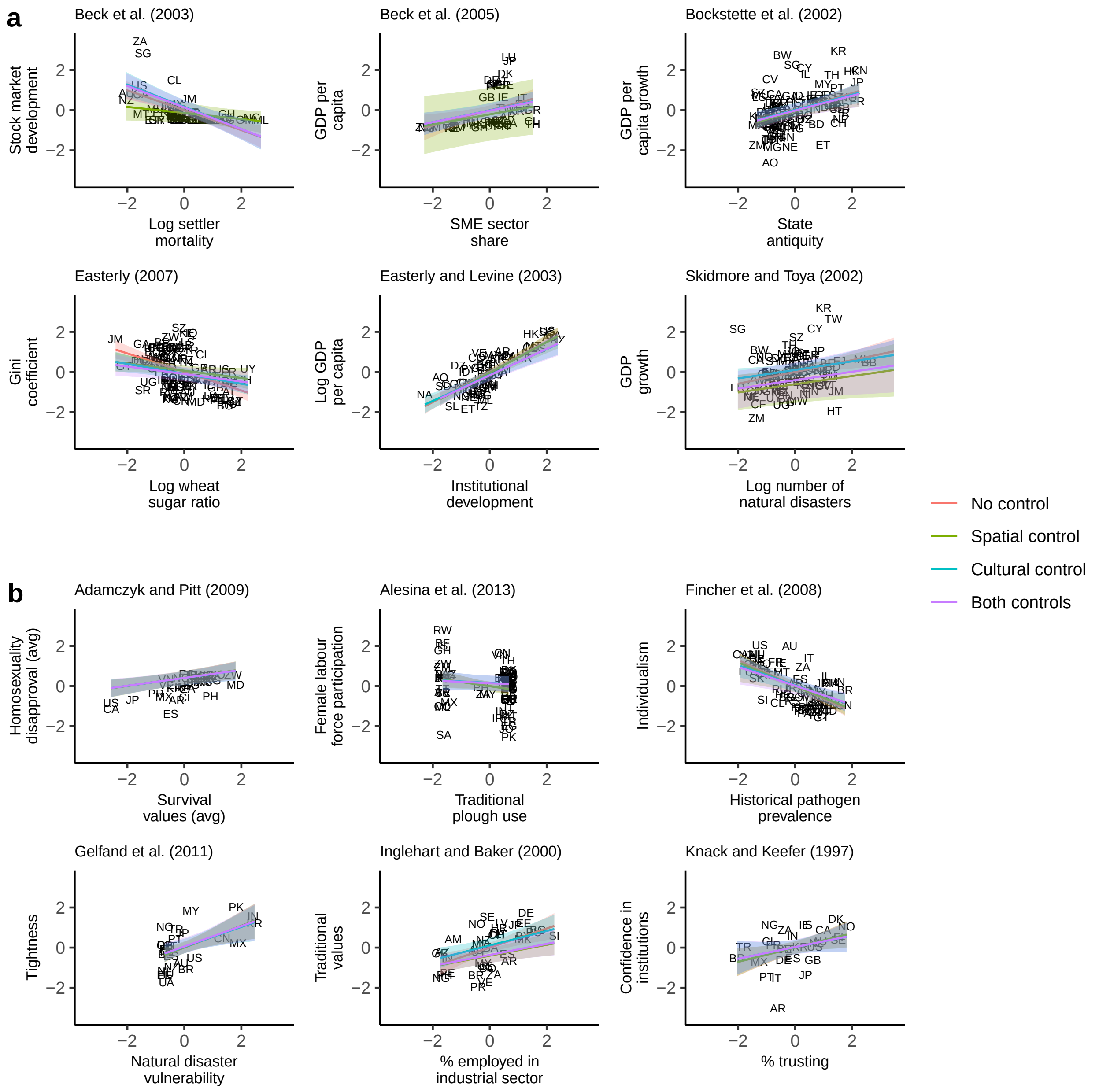

Supplementary Figure 11. Reanalysis models fitted to raw data, for economic development (a) and cultural values (b) studies. Data points are labelled using ISO 3166-1 alpha-2 letter country codes. In all reanalyses, outcome and predictor variables are standardised, making regression slopes comparable to Pearson's correlation coefficients. Lines and shaded areas represent posterior median regression lines and equal-tailed 95% credible intervals. Colours indicate predictions from models with no control (red), spatial control only (green), cultural control only (blue), or both spatial and cultural control (purple). For models with covariates (Adamzyck and Pitt 2009; Gelfand et al. 2011), marginal effects are presented holding all covariates at zero or their reference categories. GDP = gross domestic product; SME = small and medium-sized enterprise.

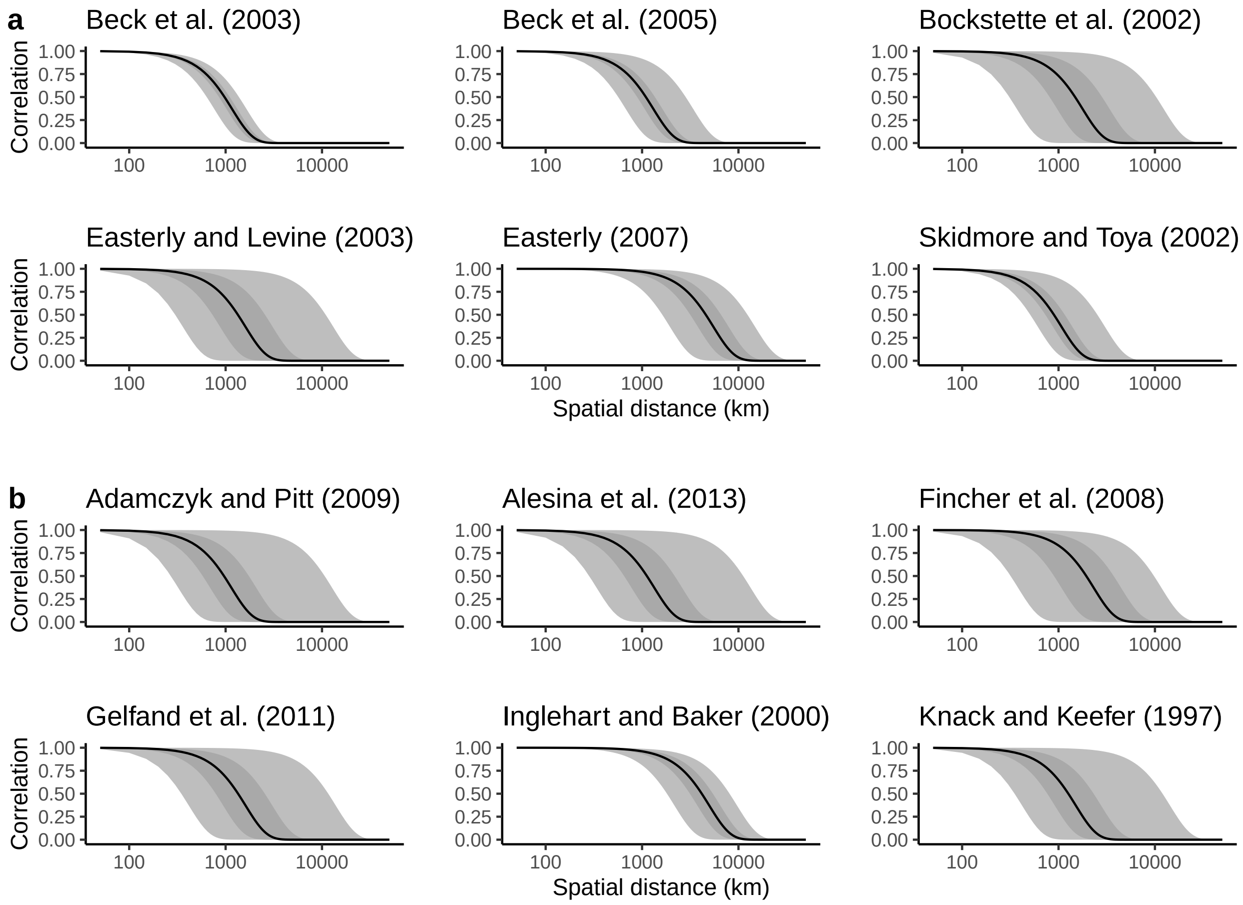

Supplementary Figure 12. Posterior estimates of Gaussian process functions mapping spatial autocorrelation onto geographic distance from our reanalyses of economic development (a) and cultural values (b) studies. The y-axis represents the amount of spatial autocorrelation between data points with increasing distance between those points on the x-axis (logged distance in kilometres). Lines and shaded areas represent median posterior spatial autocorrelation functions and equal-tailed 50% and 95% credible intervals.

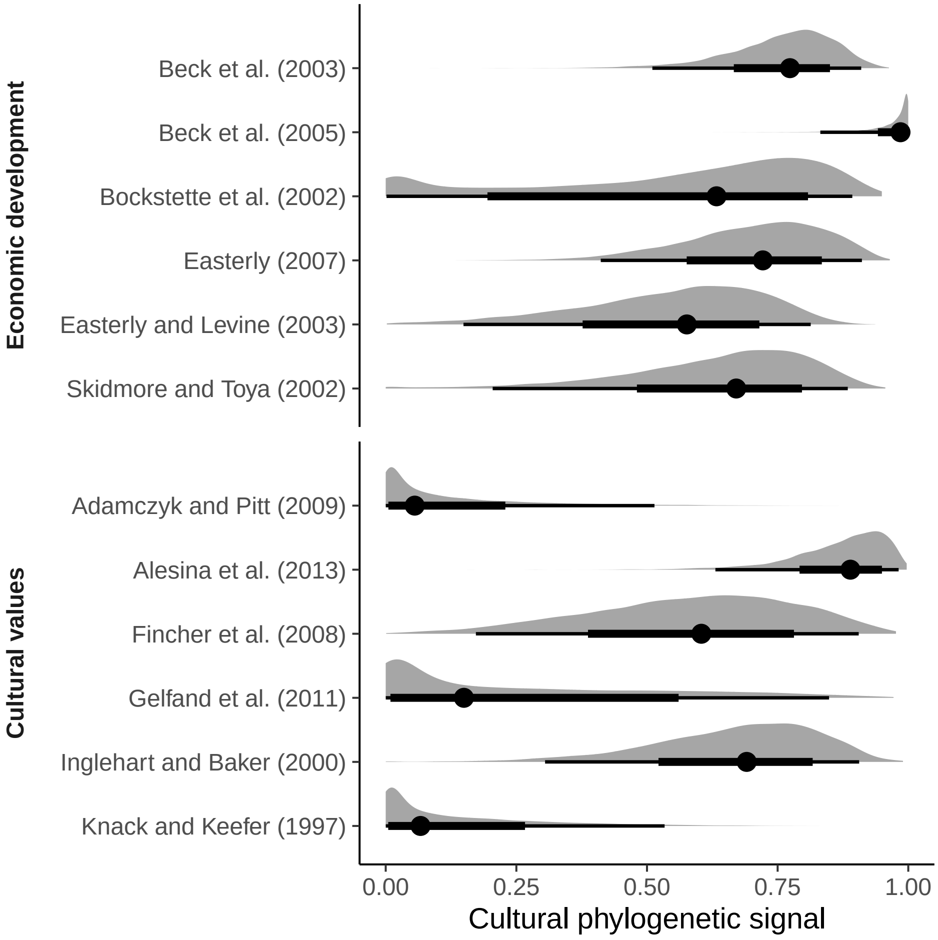

Supplementary Figure 13. Posterior estimates of cultural phylogenetic signal from our reanalyses. Cultural phylogenetic signal is operationalised as the proportion of national-level variance explained by linguistic proximity between nations. Ridges are full posterior distributions, points are posterior medians, and lines represent equal-tailed 50% and 95% credible intervals. Numbers of observations from the models are as follows, from top to bottom: n = 69, n = 45, n = 103, n = 98, n = 63, n = 89, n = 33, n = 75, n = 67, n = 28, n = 38, and n = 28.

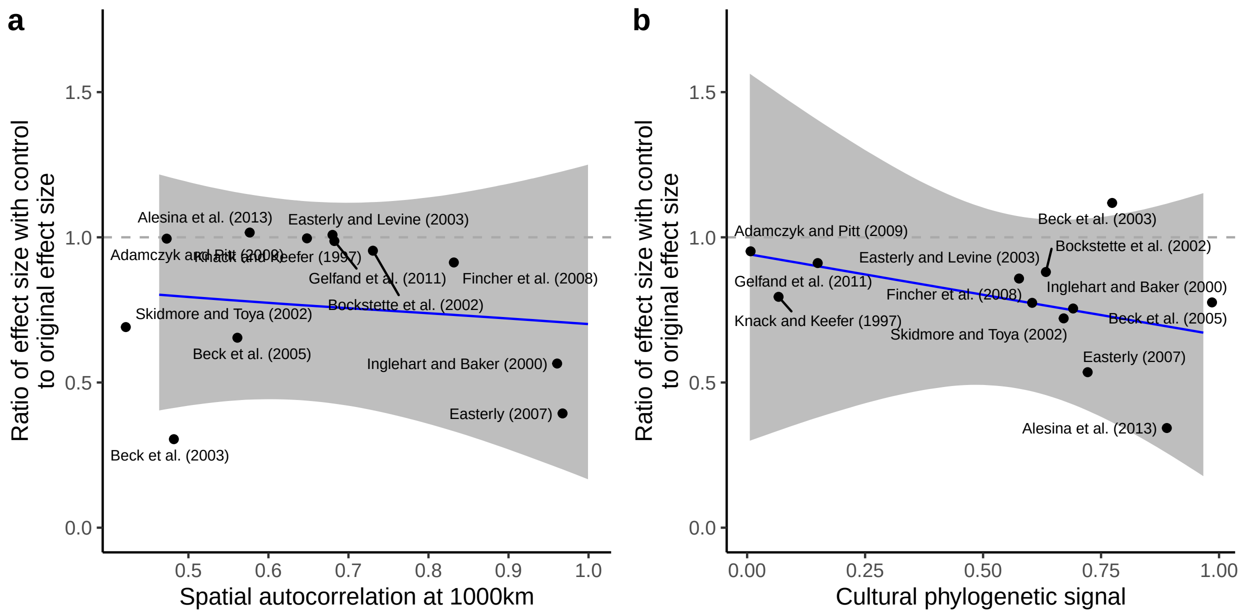

Supplementary Figure 14. *The estimated degree of spatial and cultural phylogenetic non-independence predicts reductions in effect size in our reanalysis.* (a) Higher estimated degrees of spatial autocorrelation at 1,000 km distance predict more pronounced reductions in effect sizes when controlling for non-independence. (b) Higher estimated levels of cultural phylogenetic signal predict more pronounced reductions in effect sizes when controlling for non-independence. In both panels, the y-axis represents the ratio of the effect size when controlling for spatial and cultural non-independence to the original effect size (from naive regression model), and the x-axis represents posterior median model estimates. Regression lines are plotted with equal-tailed 95% credible intervals.

Supplementary Tables

Supplementary Table 1

Geographic and cultural phylogenetic signal results for economic development and cultural values variables. Signal estimates reflect the proportion of national-level variance explained by geographic and linguistic covariance matrices. Bayes Factors (BF) reflect support for the hypothesis that the signal estimate differs from zero. HDI = Human Development Index; GDPpc = gross domestic product per capita.

Outcome	Geographic signal	Cultural phylogenetic signal
HDI	0.37, 95% CI [0.18, 0.59], BF > 100	0.62, 95% CI [0.40, 0.80], BF > 100
GDPpc	0.42, 95% CI [0.20, 0.66], BF > 100	0.56, 95% CI [0.33, 0.78], BF > 100
GDPpc growth	0.65, 95% CI [0.09, 0.98], BF = 16.79	0.26, 95% CI [0.00, 0.70], BF = 1.16
Gini index	0.74, 95% CI [0.48, 0.97], BF > 100	0.25, 95% CI [0.02, 0.51], BF = 3.97
Traditional values	0.44, 95% CI [0.17, 0.76], BF > 100	0.54, 95% CI [0.23, 0.79], BF > 100
Survival values	0.20, 95% CI [0.01, 0.45], BF = 1.96	0.78, 95% CI [0.53, 0.96], BF > 100
Tightness	0.09, 95% CI [0.00, 0.32], BF = 0.33	0.84, 95% CI [0.61, 0.97], BF > 100
Individualism	0.26, 95% CI [0.00, 0.66], BF = 1.97	0.69, 95% CI [0.29, 0.96], BF > 100

Supplementary Table 2

False positive rates in simulation with varying levels of spatial autocorrelation for the predictor variable (ρ) and for the outcome variable (λ), assuming that the true correlation is 0. Numbers represent the total number of analyses, out of 100, that estimated a slope with a two-tailed 95% confidence / credible interval excluding zero, falsely inferring a relationship when none is present.

	$\rho = 0.2$			$\rho = 0.5$			$\rho = 0.8$		
Model	$\lambda = 0.2$	$\lambda = 0.5$	$\lambda = 0.8$	$\lambda = 0.2$	$\lambda = 0.5$	$\lambda = 0.8$	$\lambda = 0.2$	$\lambda = 0.5$	$\lambda = 0.8$
No control	18	27	41	27	48	61	40	61	77
Latitude	19	25	39	25	44	60	33	58	71
Longitude	14	20	27	22	34	48	29	49	62
Continent	10	13	16	13	18	24	13	25	35
Language family	12	17	29	20	33	46	27	48	58
Mean 2000km radius	9	12	19	5	9	13	5	5	6
Conley SEs spatial	13	13	14	18	23	25	22	37	43
Conley SEs genetic	17	22	27	20	34	51	28	49	66
Bayesian spatial	14	13	11	13	15	12	16	17	23
Bayesian linguistic	12	20	21	21	26	39	28	42	49
Bayesian spatial & linguistic	11	13	9	14	13	12	17	18	21

Supplementary Table 3

False positive rates in simulation with varying levels of cultural phylogenetic autocorrelation for the predictor variable (ρ) and for the outcome variable (λ), assuming that the true correlation is 0. Numbers represent the total number of analyses, out of 100, that estimated a slope with a two-tailed 95% confidence / credible interval excluding zero, falsely inferring a relationship when none is present.

	$\rho = 0.2$			$\rho = 0.5$			$\rho = 0.8$		
Model	$\lambda = 0.2$	$\lambda = 0.5$	$\lambda = 0.8$	$\lambda = 0.2$	$\lambda = 0.5$	$\lambda = 0.8$	$\lambda = 0.2$	$\lambda = 0.5$	$\lambda = 0.8$
No control	7	14	15	8	19	30	15	26	36
Latitude	7	10	16	10	19	27	15	22	35
Longitude	5	12	14	9	16	26	13	26	35
Continent	5	6	10	3	11	21	8	20	29
Language family	7	9	12	9	14	22	10	20	32
Mean 2000km radius	5	8	12	2	12	21	4	16	25
Conley SEs spatial	2	6	7	2	9	15	6	14	20
Conley SEs genetic	5	11	13	8	13	21	8	17	26
Bayesian spatial	7	12	12	9	17	25	14	24	32
Bayesian linguistic	5	9	11	6	7	7	4	5	5
Bayesian spatial & linguistic	3	10	11	5	8	8	4	5	5

Supplementary Table 4

Estimated cross-national correlations from our reanalysis of twelve previous cross-national analyses. Numbers are posterior median slopes (equivalent to Pearson's r correlation coefficients) with equal-tailed 95% credible intervals.

Analysis	No control	Spatial control	Cultural control	Both controls
Adamczyk and Pitt (2009)	0.20, 95% CI [0.07, 0.33]	0.20, 95% CI [0.07, 0.34]	0.19, 95% CI [0.05, 0.33]	0.20, 95% CI [0.06, 0.33]
Alesina et al. (2013)	-0.21, 95% CI [-0.42, 0.01]	-0.21, 95% CI [-0.43, 0.01]	-0.07, 95% CI [-0.26, 0.11]	-0.07, 95% CI [-0.25, 0.11]
Beck et al. (2003)	-0.50, 95% CI [-0.68, -0.30]	-0.15, 95% CI [-0.38, 0.02]	-0.56, 95% CI [-0.77, -0.33]	-0.54, 95% CI [-0.76, -0.30]
Beck et al. (2005)	0.38, 95% CI [0.12, 0.64]	0.25, 95% CI [-0.01, 0.50]	0.29, 95% CI [0.10, 0.49]	0.29, 95% CI [0.09, 0.48]
Bockstette et al. (2002)	0.43, 95% CI [0.25, 0.59]	0.41, 95% CI [0.19, 0.59]	0.37, 95% CI [0.18, 0.57]	0.37, 95% CI [0.15, 0.56]
Easterly and Levine (2003)	0.75, 95% CI [0.60, 0.90]	0.76, 95% CI [0.57, 0.93]	0.65, 95% CI [0.47, 0.82]	0.66, 95% CI [0.47, 0.84]
Easterly (2007)	-0.46, 95% CI [-0.63, -0.29]	-0.18, 95% CI [-0.36, 0.01]	-0.25, 95% CI [-0.41, -0.09]	-0.19, 95% CI [-0.36, -0.01]
Fincher et al. (2008)	-0.64, 95% CI [-0.82, -0.46]	-0.59, 95% CI [-0.80, -0.21]	-0.50, 95% CI [-0.71, -0.27]	-0.47, 95% CI [-0.69, -0.18]
Gelfand et al. (2011)	0.54, 95% CI [0.20, 0.85]	0.54, 95% CI [0.19, 0.86]	0.49, 95% CI [0.14, 0.83]	0.50, 95% CI [0.15, 0.83]
Inglehart and Baker (2000)	0.48, 95% CI [0.21, 0.74]	0.27, 95% CI [0.11, 0.44]	0.36, 95% CI [0.11, 0.60]	0.28, 95% CI [0.10, 0.46]
Knack and Keefer (1997)	0.36, 95% CI [0.05, 0.67]	0.36, 95% CI [0.04, 0.66]	0.28, 95% CI [-0.10, 0.63]	0.29, 95% CI [-0.09, 0.62]
Skidmore and Toya (2002)	0.30, 95% CI [0.10, 0.49]	0.20, 95% CI [0.00, 0.41]	0.21, 95% CI [0.03, 0.40]	0.21, 95% CI [0.01, 0.41]

Supplementary Table 5

List of sample size deviations between original analyses and reanalyses.

Analysis	Original N	Reanalysis N	Reason for deviation
Adamczyk and Pitt (2009)	33	33	N/A
Alesina et al. (2013)	>177	75	Original analysis used imputed values for traditional plough use which were unavailable to us. For our reanalysis, we collected data from Tables A4 and A9 and ran our regression on the 75 nations with available data for female labour force participation in 2000 and traditional plough use.
Beck et al. (2003)	70	69	We removed one nation (Zaire) as it was not present in our linguistic distance matrix.
Beck et al. (2005)	45	45	N/A
Bockstette et al. (2002)	94	103	After manually linking state history data from Appendix A with world development indicators, we had complete data for 103 nations.
Easterly and Levine (2003)	72	63	After manually linking log GDP per capita 1995 data from Appendix Table A2 in Acemoglu et al. (2001) with an institutions index self-constructed from world governance indicators, we only had complete data for 63 nations.
Easterly (2007)	118	98	After manually linking log wheat-sugar ratio data from Appendix A with longitudinal Gini data, we only had complete data for 98 nations.
Fincher et al. (2008)	68	67	We combined England and Northern Ireland into a single nation (United Kingdom) for the reanalysis.
Gelfand et al. (2011)	30	28	We combined East and West Germany into a single nation (Germany) and removed a high leverage point with a substantially higher disaster rate than other nations (Venezuela).
Inglehart and Baker (2000)	65	38	After manually linking traditional values data from the World Values Survey with data on percentage total employment in industry from World Development Indicators, we only had complete data for 38 nations.
Knack and Keefer (1997)	28	28	N/A
Skidmore and Toya (2002)	89	89	N/A

Supplementary References

1. Bürkner, P.-C. brms: An R package for Bayesian multilevel models using Stan. *Journal of Statistical Software* **80**, 1–28 (2017).
