# Supplementary material for: Cross-national analyses require additional controls to account for the non-independence of nations
Source: Nat Commun. 2023 Sep 18;14:5776. doi: 10.1038/s41467-023-41486-1 (PMC10507061; doi:10.1038/s41467-023-41486-1)
Supplement: Supplementary file 3 — Description of Additional Supplementary Files [file 41467_2023_41486_MOESM3_ESM.pdf]

Description of Additional Supplementary Files

File Name: Supplementary Data 1
Description: Data file for literature review, including data, codebook, instructions for the literature review, Web of Science queries, raw exports from Web of Science for values review, and raw exports from Web of Science for economic development review.
