# Supplementary material for: Cross-national analyses require additional controls to account for the non-independence of nations
Source: Nat Commun. 2023 Sep 18;14:5776. doi: 10.1038/s41467-023-41486-1 (PMC10507061; doi:10.1038/s41467-023-41486-1)
Supplement: Supplementary file 5 — Reporting Summary [file 41467_2023_41486_MOESM5_ESM.pdf]

nature portfolio

Corresponding author(s): Scott Claessens and Quentin D. Atkinson

Last updated by author(s): Aug 8, 2023

# Reporting Summary

Nature Portfolio wishes to improve the reproducibility of the work that we publish. This form provides structure for consistency and transparency in reporting. For further information on Nature Portfolio policies, see our Editorial Policies and the Editorial Policy Checklist.

## Statistics

For all statistical analyses, confirm that the following items are present in the figure legend, table legend, main text, or Methods section.

| n/a | Confirmed | |
|---|---|---|
| ☐ | ☒ | The exact sample size (*n*) for each experimental group/condition, given as a discrete number and unit of measurement |
| ☐ | ☒ | A statement on whether measurements were taken from distinct samples or whether the same sample was measured repeatedly |
| ☐ | ☒ | The statistical test(s) used AND whether they are one- or two-sided<br>*Only common tests should be described solely by name; describe more complex techniques in the Methods section.* |
| ☐ | ☒ | A description of all covariates tested |
| ☐ | ☒ | A description of any assumptions or corrections, such as tests of normality and adjustment for multiple comparisons |
| ☐ | ☒ | A full description of the statistical parameters including central tendency (e.g. means) or other basic estimates (e.g. regression coefficient) AND variation (e.g. standard deviation) or associated estimates of uncertainty (e.g. confidence intervals) |
| ☐ | ☒ | For null hypothesis testing, the test statistic (e.g. *F*, *t*, *r*) with confidence intervals, effect sizes, degrees of freedom and *P* value noted<br>*Give P values as exact values whenever suitable.* |
| ☐ | ☒ | For Bayesian analysis, information on the choice of priors and Markov chain Monte Carlo settings |
| ☐ | ☒ | For hierarchical and complex designs, identification of the appropriate level for tests and full reporting of outcomes |
| ☐ | ☒ | Estimates of effect sizes (e.g. Cohen's *d*, Pearson's *r*), indicating how they were calculated |

*Our web collection on statistics for biologists contains articles on many of the points above.*

## Software and code

Policy information about availability of computer code

| | |
|---|---|
| Data collection | We used R version 4.2.1., the brms R package (version 2.18.0), and the geosphere R package (version 1.5-14) to simulate data. R code used to simulate data can be found on GitHub (https://github.com/ScottClaessens/crossNationalCorrelations). All code is embedded in a reproducible pipeline using the targets R package (version 0.13.1) and used to generate the final manuscript using the papaja R package (version 0.1.1). For more information on package versions, see "sessionInfo.txt" on GitHub. |
| Data analysis | We used R version 4.2.1., the brms R package (version 2.18.0), and the conleyreg R package (version 0.1.7) to analyse the data. R code used to analyse data can be found on GitHub (https://github.com/ScottClaessens/crossNationalCorrelations). All code is embedded in a reproducible pipeline using the targets R package (version 0.13.1) and used to generate the final manuscript using the papaja R package (version 0.1.1). For more information on package versions, see "sessionInfo.txt" on GitHub. |

For manuscripts utilizing custom algorithms or software that are central to the research but not yet described in published literature, software must be made available to editors and reviewers. We strongly encourage code deposition in a community repository (e.g. GitHub). See the Nature Portfolio guidelines for submitting code & software for further information.

## Data

Policy information about availability of data

All manuscripts must include a data availability statement. This statement should provide the following information, where applicable:

- Accession codes, unique identifiers, or web links for publicly available datasets
- A description of any restrictions on data availability
- For clinical datasets or third party data, please ensure that the statement adheres to our policy

All data are publicly available on GitHub (https://github.com/ScottClaessens/crossNationalCorrelations). We used the following publicly available datasets in the

study:
- Data on human development were retrieved from the United Nations Development Programme (https://hdr.undp.org/en/content/download-data)
- Data on GDP per capita, annual GDP per capita growth, and the Gini coefficient were retrieved from the World Bank (https://data.worldbank.org/)
- Data on traditional vs. secular values and survival vs. self-expression values were retrieved from the World Values Survey (https://www.worldvaluessurvey.org/wvs.jsp)
- Data on cultural tightness were retrieved from the OSF repository for Gelfand et al. 2021 (https://osf.io/47pe8/)

Other datasets (e.g., for replications) were retrieved from tables and supplementary tables directly from the papers cited in the main text. These have been made publicly available on GitHub (https://github.com/ScottClaessens/crossNationalCorrelations).

# Field-specific reporting

Please select the one below that is the best fit for your research. If you are not sure, read the appropriate sections before making your selection.

☐ Life sciences ☒ Behavioural & social sciences ☐ Ecological, evolutionary & environmental sciences

For a reference copy of the document with all sections, see nature.com/documents/nr-reporting-summary-flat.pdf

# Behavioural & social sciences study design

All studies must disclose on these points even when the disclosure is negative.

| | |
|---|---|
| Study description | Quantitative, simulation and re-analysis study. |
| Research sample | The Integrated World Values Survey collects data from representative samples across 116 nations (https://www.worldvaluessurvey.org/wvs.jsp). Data on cultural tightness (57 nations) and individualism (97 nations) were taken from previous research, drawn from non-representative samples (see Methods). Human Development Index (HDI) data for 189 nations were downloaded from https://hdr.undp.org/en/content/download-data. Other national-level data on economic development (n = 167-209 nations) were taken from the World Bank (https://data.worldbank.org/). The rationale for using these datasets is that they are the largest datasets available for the variables of interest, with the most comprehensive cross-national coverage. Simulated datasets were also generated by the first author. Data from twelve previous cross-national studies in economics and psychology were collated, and these datasets can be found here: https://github.com/ScottClaessens/crossNationalCorrelations |
| Sampling strategy | The World Values Survey uses full probability or a combination of probability and stratified sampling methods. HDI is a national-level measure that does not require participant sampling. For the twelve previous cross-national studies, researchers used the largest sample of nations available to them. We used all available data in our analyses, and no sample size calculations were performed. |
| Data collection | The World Values Survey collects data via face-to-face interviews or paper questionnaires where only participants and researchers are present. All cross-national data were collected from existing public sources. Researchers collating data were not aware of the hypotheses tested in this study. |
| Timing | The World Values Survey has collected data since 1981. HDI values are available from 1990, while data from the World Bank are available from 1960. We do not know exact start and stop dates of data collection since these are secondary data sources. |
| Data exclusions | No data were excluded from analyses. |
| Non-participation | We did not collect primary data on participants in this study, and so no participants dropped out or were excluded. |
| Randomization | No randomization was used in our study. All analyses reported in our study are correlational, with some analyses controlling for additional covariates. |

# Reporting for specific materials, systems and methods

We require information from authors about some types of materials, experimental systems and methods used in many studies. Here, indicate whether each material, system or method listed is relevant to your study. If you are not sure if a list item applies to your research, read the appropriate section before selecting a response.

## Materials & experimental systems

| n/a | Involved in the study | |
|---|---|---|
| ☒ | ☐ | Antibodies |
| ☒ | ☐ | Eukaryotic cell lines |
| ☒ | ☐ | Palaeontology and archaeology |
| ☒ | ☐ | Animals and other organisms |
| ☒ | ☐ | Human research participants |
| ☒ | ☐ | Clinical data |
| ☒ | ☐ | Dual use research of concern |

## Methods

| n/a | Involved in the study | |
|---|---|---|
| ☒ | ☐ | ChIP-seq |
| ☒ | ☐ | Flow cytometry |
| ☒ | ☐ | MRI-based neuroimaging |
